# Draft Genome Sequence of a Fluconazole-Resistant *Candida auris* Strain from a Candidemia Patient in India

**DOI:** 10.1128/genomeA.00722-15

**Published:** 2015-07-16

**Authors:** Cheshta Sharma, Nitin Kumar, Jacques F. Meis, Rajesh Pandey, Anuradha Chowdhary

**Affiliations:** a Department of Medical Mycology, Vallabhbhai Patel Chest Institute, University of Delhi, Delhi, India; b Host-Microbiota Interactions Laboratory, Wellcome Trust Sanger Institute, Hinxton, United Kingdom; c Department of Medical Microbiology and Infectious Diseases, Canisius-Wilhelmina Hospital, Nijmegen, The Netherlands; d Department of Medical Microbiology, Radboudumc, Nijmegen, The Netherlands; e CSIR Ayurgenomics Unit-TRISUTRA, Council of Scientific & Industrial Research–Institute of Genomics and Integrative Biology (CSIR-IGIB), New Delhi, India

## Abstract

*Candida auris* is a multidrug-resistant yeast incriminated in a wide spectrum of invasive infections, especially in intensive care settings. The first draft genome sequence of *C. auris*, VPCI 479/P/13, from a case with fungemia was sequenced using the Illumina MiSeq platform. The estimated genome size is 12.3 Mb, with 6,675 coding sequences.

## GENOME ANNOUNCEMENT

*Candida auris* is a multidrug-resistant yeast in the *Metschnikowiaceae* clade, which was first reported in 2009 from the external auditory canal of a Japanese patient (1). In the last 5 years, a wide spectrum of clinical manifestations due to this unusual yeast, ranging from fungemia to deep-seated infections with high mortality rates, have been reported (2). Further, multidrug-resistant clonal strains of *C. auris* are widespread in hospitals, suggesting nosocomial transmission (2, 3). Recent reports of this pathogen from Kuwait, India, South Korea, and South Africa highlight the problem of increasing treatment failures in infections due to *C. auris* (3–6). *Candida auris* is often misidentified by commercial automated systems as *Candida haemulonii*, and it is mainly resistant to fluconazole and exhibits elevated MICs to voriconazole, amphotericin B, and caspofungin (2, 7). Thus, the accurate identification of *C. auris* by molecular methods and antifungal susceptibility testing using reference methods is of paramount importance. The lack of whole-genome data of *C. auris* prompted us to undertake the draft genome sequencing of *C. auris* VPCI 479/P/13 obtained from a blood culture from a patient with fungemia in Delhi, India. The isolate was identified by amplification and sequencing of the *ITS*, *D1D2*, *RPB1*, and *RPB2* genes and showed 99% homology with South Korean *C. auris* isolates (KCTC 17809 and KCTC 17810). The isolate was resistant to fluconazole with an MIC of >64 µg/ml.

Genomic DNA was extracted using a column-based method with the QIAamp DNA minikit (Qiagen, Hilden, Germany). One nanogram of genomic DNA (gDNA) in the Nextera XT DNA sample preparation protocol (Illumina, Inc., San Diego, CA, USA) was used to generate sequencing-ready libraries, according to the manufacturer’s instructions. The sequencing library was quantified using Qubit 2.0 (Invitrogen, Waltham, MA, USA) and qualified by Bioanalyzer (Agilent Technologies, Richardson, TX, USA). The genome of *C. auris* VPCI 479/P/13 was sequenced using the Illumina MiSeq platform with a MiSeq version 3 protocol (paired end, 300 × 2 bp). The adaptor contamination was removed from the reads, followed by trimming from both sides using the modified Mott trimming algorithm to reach a Q30 score. The FASTQ files were then imported in the CLC Genomics Workbench software (CLC bio-Qiagen). Genome assembly was achieved by the combination of Velvet version 1.2.08 (8), SSPACE 2.0, and GapFiller version 1.10 (9, 10). Repetitive sequences were masked using RepeatMasker version 4.0.5 (http://www.repeatmasker.org), followed by *ab initio* gene prediction using GeneMark-ES 2.0 (11). Next, rRNAs and tRNAs were predicted with RNAmmer and tRNAscan-SE version 1.21, respectively (12, 13). The draft genome of *C. auris* VPCI 479/P/13 is 12.3 Mb, with a G+C content of 44.8%, distributed on 533 scaffolds (≥1,000 bp) with an *N*_50_ length of 37,205 bp. The complete genome sequence of *C. auris* VPCI 479/P/13 contains 6,675 coding sequences (CDSs), one 5.8S rRNA, 184 tRNAs, and 3,262 repetitive elements. The draft genome sequence presented here will facilitate further genomic studies on the biology and virulence of *C. auris*.

### Nucleotide sequence accession number. 

The draft genome sequence of *C. auris* strain VPCI 479/P/13 has been deposited at ENA under accession number CVRJ00000000.
